# CT-based 3D morphometric reference data on osseous TMJ subunits in a European cohort: relevance for patient-specific implant research

**DOI:** 10.1186/s12903-026-10090-2

**Published:** 2026-10-10

**Authors:** Sönke Maximilian Braß, Christoph Schinzel, Rijahd Sahmer Boustani, Max Lukas Linderkamp, Michael-Tobias Neuhaus, Fritjof Lentge, Nils-Claudius Gellrich, Philippe Korn, Philipp Jehn

**Affiliations:** 1 Department of Oral and Maxillofacial Surgery, Hannover Medical School, Carl Neuberg Str. 1, Hannover, 30625 Germany; 2 Department of Ophthalmology, University Hospital Göttingen, Robert- Koch-Str. 40, Göttingen, 37075 Germany

**Keywords:** TMJ, Temporomandibular joint, Mandibular condyle, Glenoid fossa, Patient-specific implant

## Abstract

**Background:**

Patient-specific alloplastic prostheses are increasingly used for reconstruction of the temporomandibular joint (TMJ). However, the extent to which articulating implant components should reflect native osseous TMJ morphology remains unresolved. This study used CT-derived three-dimensional (3D) virtual-digital surface models to analyze osseous TMJ subunits in humans and provide morphometric reference data for future patient-specific TMJ implant research.

**Methods:**

In this retrospective exploratory study, 97 computed tomography (CT) scans from Hannover Medical School were analyzed. High-quality 3D virtual-digital models of the articulating TMJ subunits, including the mandibular condyle and glenoid fossa, were generated and used to measure relevant TMJ parameters. Parameters were compared according to sex and age, and correlation analyses were performed.

**Results:**

Morphometric variability was observed among the osseous TMJ subunits. Men showed larger medial-lateral diameters of the mandibular condyle, greater articular tubercle height, and larger zygomatic arch root dimensions. Women showed a larger anterior-posterior diameter of the glenoid fossa. Middle-aged participants showed a larger anterior-posterior diameter of the mandibular condyle and larger sagittal dimensions of the zygomatic arch root compared with young-aged participants. Correlation analysis revealed associations between selected parameters, particularly between zygomatic arch root diameter and articular tubercle height.

**Conclusion:**

This study provides CT-based 3D morphometric reference data on osseous TMJ subunits in a European cohort. The findings indicate inter-individual morphometric variability and exploratory sex- and age-related differences in selected parameters. These data may provide anatomical reference information for future patient-specific TMJ implant research. However, the present study does not evaluate implant fit, mandibular kinematics, biomechanical performance, or clinical outcomes. Therefore, the translational relevance of these findings requires further validation.

**Supplementary Information:**

The online version contains supplementary material available at https://doi.org/10.1186/s12903-026-10090-2.

## Background

Advancements in digital technology allow for more individualized treatment approaches in oral and maxillofacial surgery. In temporomandibular joint (TMJ) reconstruction, both stock and patient-specific alloplastic prostheses are used when conservative or minimally invasive treatment options have been exhausted or are insufficient to address clinically and morphologically relevant TMJ pathology [1–4]. Patient-specific TMJ prostheses aim to improve anatomical adaptation to the individual skeletal situation, particularly at the bone-facing and fixation surfaces of the skull base and mandibular ramus [5, 6]. However, the extent to which the articulating components themselves should reproduce native TMJ morphology remains a complex and unresolved question. Current implant systems do not necessarily aim to replicate natural articular geometry exactly, as prosthesis design must also consider biomechanical and engineering requirements such as stability, material properties, implant safety and manufacturability, and long-term survivorship [5, 7, 8]. Therefore, morphometric data on native osseous TMJ morphology should be interpreted as anatomical reference information rather than as direct evidence for improved prosthetic function. Nevertheless, detailed knowledge of the natural osseous morphology of the articulating TMJ subunits, including the mandibular condyle and glenoid fossa, may be relevant for future implant-related research. Previous CT based studies have commonly performed morphometric measurements directly on radiological image datasets or multiplanar reconstructions [9]. In contrast, the present study first generated high-quality virtual-digital 3D surface models from CT data and subsequently performed all measurements on these standardized models. The primary hypothesis of this study was that the osseous articulating TMJ subunits show measurable inter-individual morphometric variability in a European cohort. Secondary exploratory hypotheses were that selected TMJ parameters differ according to sex and age and that correlations exist between condylar, glenoid fossa, and zygomatic arch-related parameters. The aim of this study was therefore to characterize the anatomical and geometric variability of the osseous articulating TMJ subunits in a European cohort using a unified 3D surface model-based morphometric workflow. By analyzing multiple condylar and glenoid fossa-related parameters, this study seeks to provide descriptive morphometric reference data with potential relevance for future patient-specific TMJ implant research.

## Materials and methods

### Data collection

In this retrospective exploratory study, a total of 97 computed tomography (CT) scans performed in the Department of Oral and Maxillofacial Surgery at Hannover Medical School between 2017 and 2020 were included for TMJ analysis. Inclusion criteria were availability of a CT scan in digital imaging and communications in medicine (DICOM) format, slice thickness between 0.5 and 1.0 mm, coverage of the proximal mandible and TMJ region, and patient age between 18 and 80 years. To ensure analysis of physiologic osseous TMJ morphology, exclusion criteria were defined. CT scans showing pathologies in the TMJ region, including fractures of the mandibular condyle, zygomatic arch root or skull base, as well as neoplasms, cysts, hyperplasia, advanced degenerative joint disease, osteoarthritis, bone resorption or ankylosis were excluded from analysis. To contextualize the adequacy of the included cohort for correlation analysis, a sample size estimation was performed based on the expected correlation coefficient. Assuming an expected moderate correlation of *r* = 0.30, a two-sided alpha level of 0.05, and a statistical power of 80%, approximately 85 participants were required. The final sample of 97 CT scans therefore exceeded the estimated sample size for detecting moderate correlations. A balanced age and sex distribution ratio was considered.

### Creation of virtual-digital 3D models

To investigate the natural osseous articulating subunits of the human TMJ (i.e., the mandibular condyle unit and the glenoid fossa unit), segmentation and surface smoothing were performed to achieve high-quality virtual-digital 3D models of the bony jaw structures from the radiographic datasets using Brainlab iPlan CMF 3.0 software (Brainlab, Munich, Germany) [10]. Hounsfield Units (HU) ranging from 272 to 3071 HU were used for selection of bony tissue to cover the typical HU spectrum of human bone [11]. Following segmentation, surface smoothing was performed using MeshLab software (ISTI-CNR, Pisa, Italy) due to considerable differences of surface quality in the segmented virtual-digital 3D-models [12]. Applying a Laplace-filter, additionally, focal points and a computational center were generated and moved into the geometric center of the investigated anatomical landmarks to achieve smooth polygon meshes in all cases. Through this, high-quality virtual-digital 3D models were created enabling comparable analysis of the defined bony jaw structures and articulating subunits.

### Measurement of TMJ parameters concerning the mandibular condyle and glenoid fossa unit

**Fig. 1:**
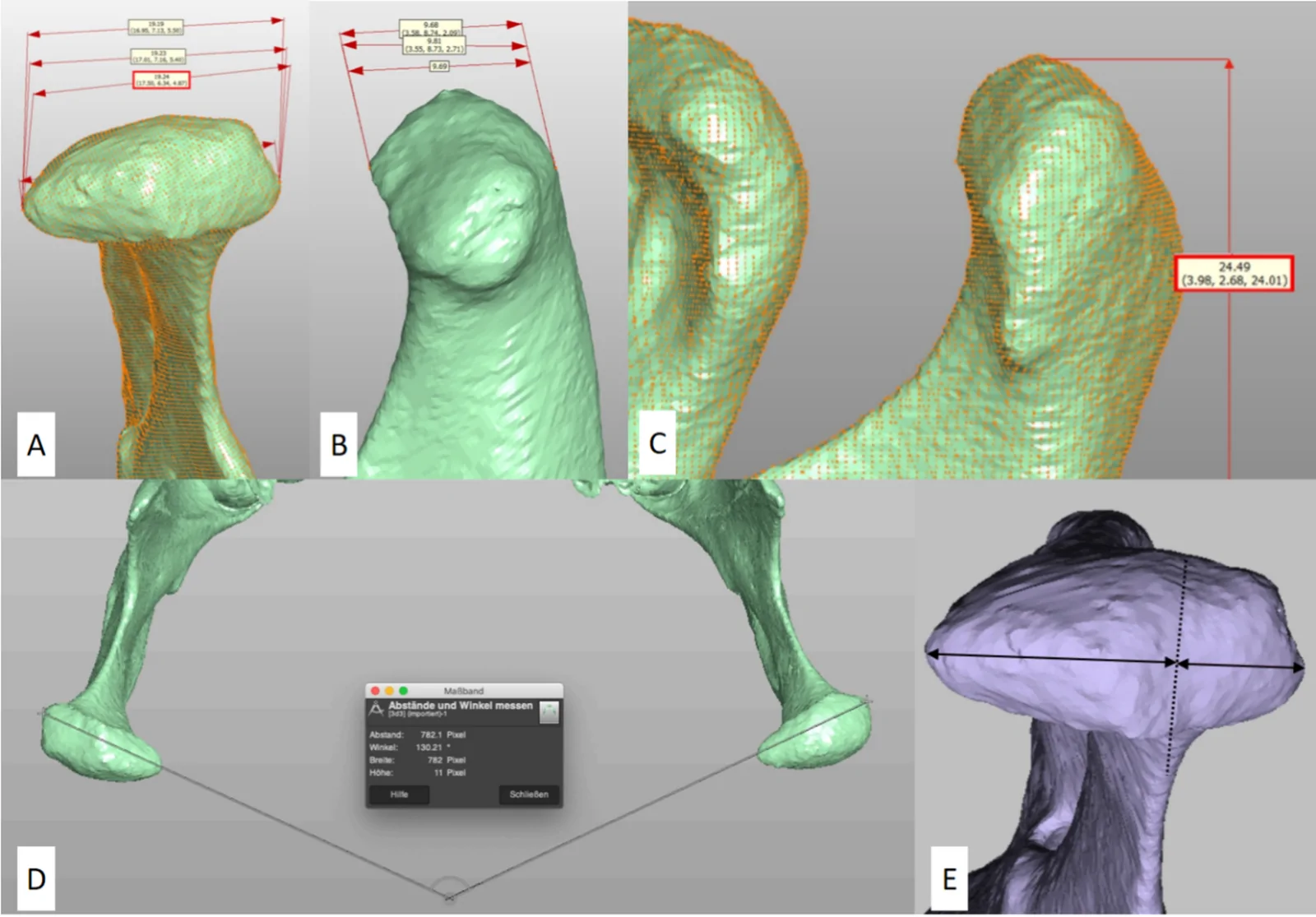
Measurement of TMJ parameters (mandibular condyle unit). **A** Condyle medial-lateral (CML). **B** Condyle anterior-posterior (CAP). **C** Condylar process height (CPH). **D** Intercondylar angle (ICA). **E** Ramus-condyle ratio (RCR)

For measurement of the relevant TMJ parameters, 3D-tool (3D-Tool GmbH & Co. KG, Weinheim, Germany) and GIMP software (Global Open-Source-Software) were used [13, 14]. Using 3D-Tool, a reliable surface topography was generated to accurately identify reproducible anatomical landmarks and measure distances and angles on the virtual-digital 3D models of the articulating TMJ subunits. For the mandibular condyle unit, firstly, largest diameter of the condyle medial-lateral (CML) and the condyle anterior-posterior (CAP) was measured in each virtual digital 3D model (Fig. 1A and B). The condylar process height (CPH) was subsequently evaluated as distance between the highest point of the mandibular condyle and the lowest point of the mandibular notch (Fig. 1C). Measurement of the intercondylar angle (ICA) was performed, additionally, by insertion of subsidiary straight lines passing the medial-lateral axis of both condylar processes (Fig. 1D). Furthermore, existing ramus-condyle ratio (RCR) was calculated from measured distances of the medial and lateral condylar pole to a virtually inserted subsidiary line extension of the mandibular neck starting from the mandibular notch to the cranial condyle side (Fig. 1E).

For the glenoid fossa unit, measurement of the defined TMJ parameters was performed using both topographic anatomical surface points and subsidiary sectional planes in the virtual-digital 3D models. For this, sectional planes were defined by software support and reproducibly placed into the virtual model.

**Fig. 2:**
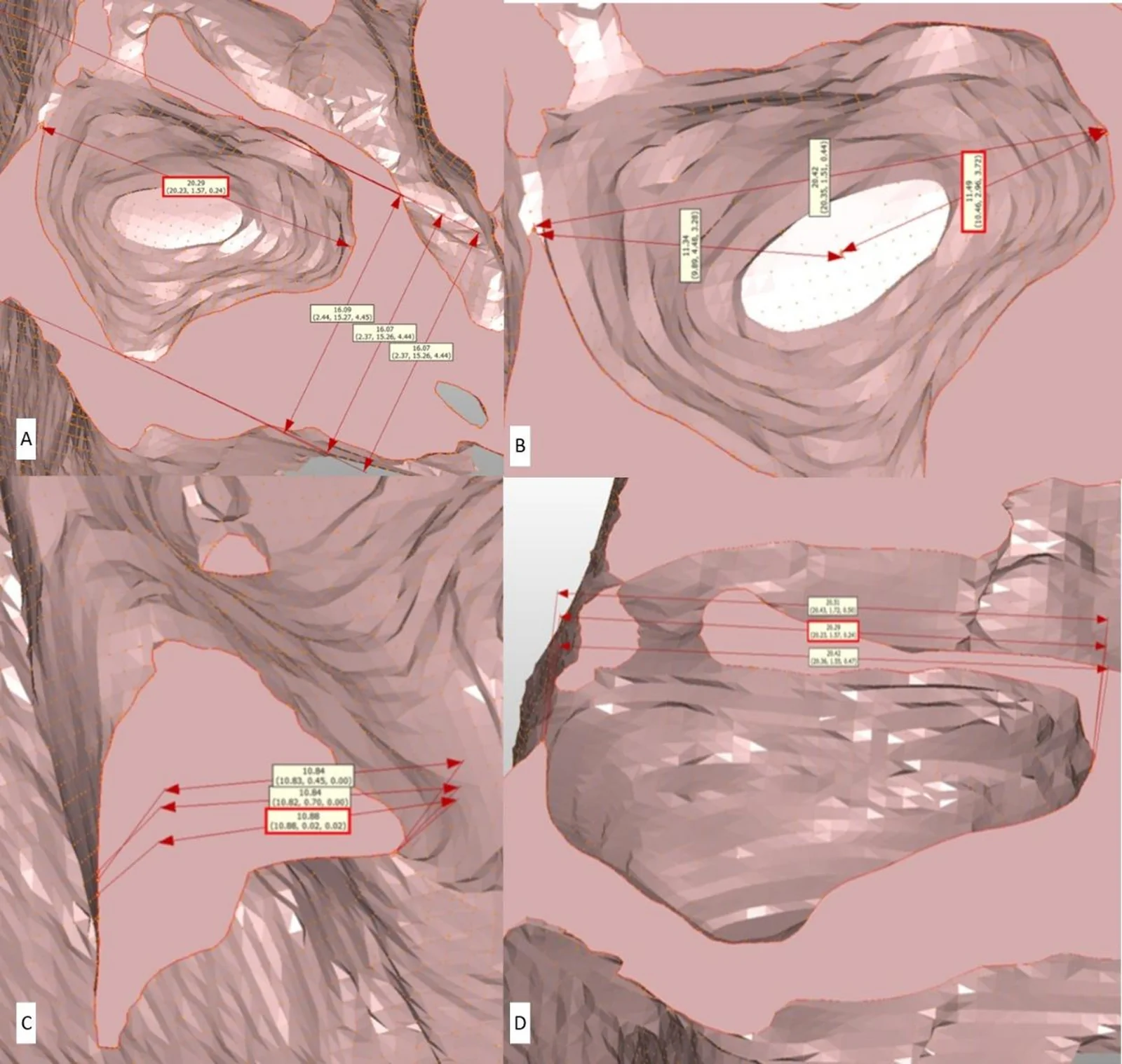
Measurement of TMJ parameters (glenoid fossa unit). **A** Fossa anterior-posterior (FAP). **B** Fossa´s deepest point (FDP). **C** Articular tubercle height (ATH). **D** Fossa medial-lateral (FML)

First, the largest diameter of the glenoid fossa anterior-posterior (FAP), the fossa´s deepest point (FDP), the temporal bone´s articular tubercle height (ATH), and the largest diameter of the fossa medial-lateral (FML) were measured. By insertion of a subsidiary horizontal sectional plane crossing the most lateral border of the glenoid fossa, FAP was measured in direction of the zygomatic arch root perpendicular to a virtual line passing the largest medial-lateral diameter of the glenoid fossa (Fig. 2A). Subsequently, FDP was calculated as distance from the deepest fossa´s point to the subsidiary horizontal plane (Fig. 2B). ATH was measured at right angle from the lowest point of the articular tubercle to the superior rim of the temporal bone´s zygomatic arch process (Fig. 2C). FML was finally captured as the distance from the glenoid fossa´s most lateral border to the most medial border in the subsidiary horizontal sectional plane (Fig. 2D).

**Fig. 3:**
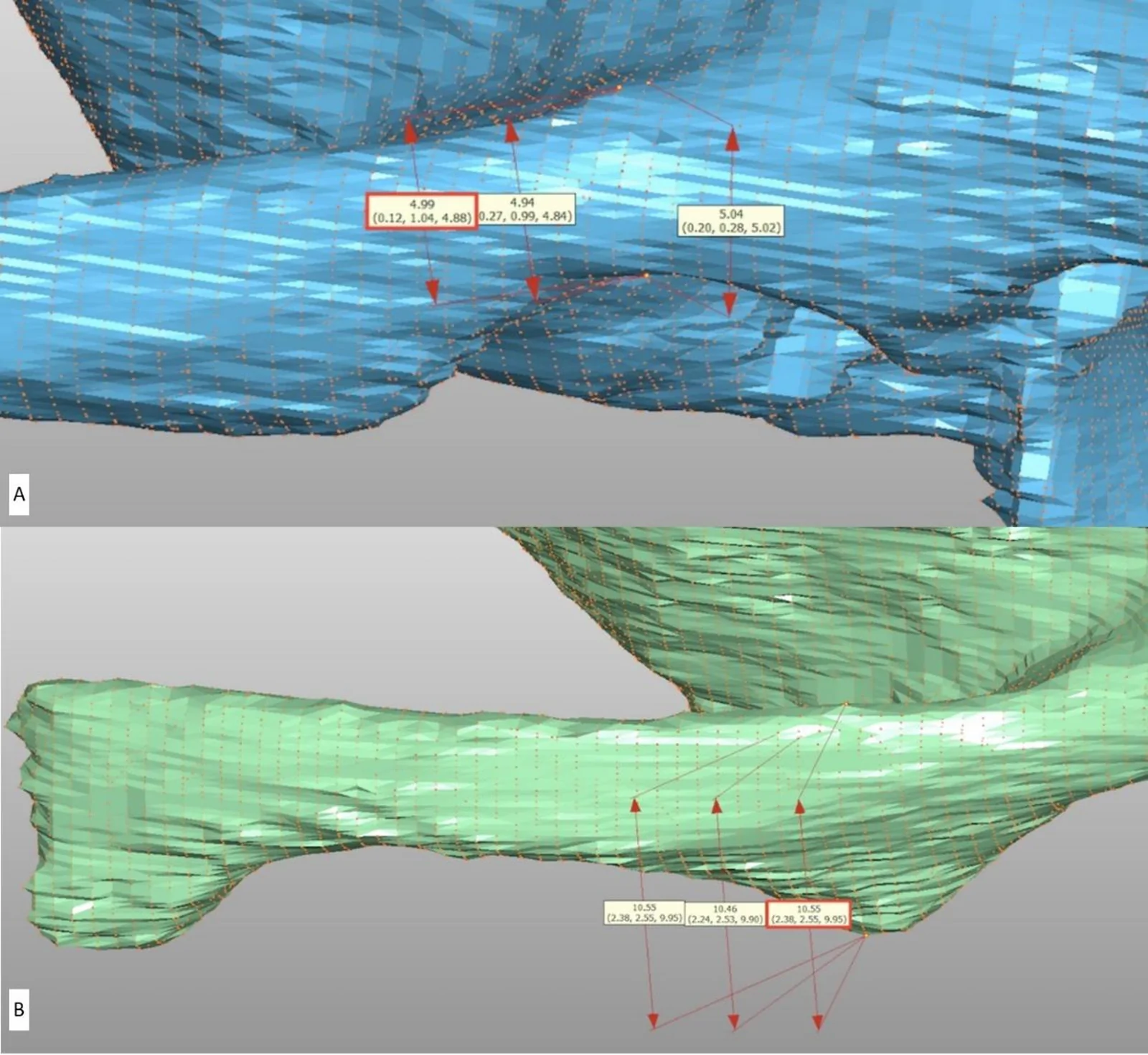
Measurement of TMJ parameters (glenoid fossa unit). **A** Zygomatic arch root sagittal (ZAS). **B** Zygomatic arch root transverse (ZAT)

Following FAP, FDP, ATH and FML measurement, secondly, glenoid fossa parameters concerning thickness of the zygomatic arch root were measured in the sagittal and transverse sectional plane. The distance between the articular tubercle and the diameter of the zygomatic arch root in the sagittal plane (TZD) was additionally captured. Regarding the thickness of the zygomatic arch root in the sagittal plane (ZAS), thickness was determined as the distance from the deepest point of the glenoid fossa´s lateral border to the superior rim of the temporal bone´s zygomatic arch process (Fig. 3A**)**. Thickness of the zygomatic arch root in the transverse plane (ZAT) was then measured as the distance from the lateral border of the zygomatic arch to the anterior border of the temporal bone´s articular tubercle (Fig. 3B). The distance between the articular tubercle and the largest diameter of the zygomatic arch root in the sagittal plane (TZD) was subsequently calculated from the measurement points used for ATH and ZAS.

For each parameter, measurements were obtained separately for the right and left TMJ. Right- and left-sided values were compared using paired statistical testing. For the main analyses, bilateral values were averaged per patient for each parameter to avoid treating both TMJs from the same individual as independent observations. To minimize intraindividual measurement variability, all parameters were measured three times by the same observer on both the left and right TMJ, and the mean value was calculated for each parameter and used for further analysis.

### Statistical analysis

Both descriptive and statistical analysis were performed to evaluate measurement and compare TMJ characteristics. For this, maximum (max) and minimum (min) values of each parameter were recorded and both mean values (mean) and standard deviations (sd) were calculated. Linear distances were recorded in millimeters (mm), angles in degrees (°), and ratios in percent (%). Student´s t-test for independent variables was used to investigate comparability between raw radiographic dataset objects and the surface smoothed digital-virtual 3D models.

Subgroup analyses were performed according to sex (male = m; female = w) and age (years). For this, analyzed human radiographic datasets were assigned to one of three age subgroups (young-aged group = 18–38 years; middle-aged group = 39–59 years; old-aged group = 60–79 years). For the analysis of sex-specific differences in mean values, Student’s t-test for independent samples was applied. Potential differences between age groups were assessed using one-way analysis of variance (ANOVA). Sex-specific subgroup analyses across age groups were additionally performed using ANOVA followed by Bonferroni-corrected post hoc tests for multiple comparisons. Correlation analysis among the measured TMJ parameters was performed using GraphPad Prism (GraphPad, San Diego, California, USA). A Pearson correlation coefficient (*r*) > 0.5 was considered to indicate a strong linear relationship between variables [15]. A *p*-value < = 0.05 was considered statistically significant.

Because this was a retrospective exploratory study, inferential analyses were performed to identify potential morphometric patterns rather than to provide confirmatory evidence. Formal testing of normality and homogeneity of variance was not included in the original statistical workflow. In addition, correction across the full family of parameter-wise sex- and age-related tests was not performed. Therefore, results from parametric tests and isolated p-values close to the conventional significance threshold should be interpreted cautiously and regarded as exploratory.

## Results

For the mandibular condyle unit, mean values were 20.64 mm for CML, 10.74 mm for CAP, and 18.91 mm for CPH. Mean ICA was 136.58°, and mean RCR was 67.15%. For the glenoid fossa unit, mean values were 19.74 mm for FML, 10.65 mm for FAP, and 4.07 mm for FDP. Additional mean values were 9.91 mm for ATH, 4.99 mm for ZAS, 10.32 mm for ZAT, and 9.53 mm for TZD (Table 1).

**Table 1:** Measurement of TMJ parameters

Mandibular condyle unit	max	min	mean	sd
CML	25.44 mm	14.84 mm	20.64 mm	2.02 mm
CAP	15.44 mm	7.54 mm	10.74 mm	1.35 mm
CPH	25.14 mm	11.40 mm	18.91 mm	2.74 mm
ICA	167.58 °	104.67 °	136.58 °	1.25 °
RCR	74.15%	57.81%	67.15%	3.11%
Glenoid fossa unit			*mean*	*sd*
FML	24.96 mm	13.02 mm	19.74 mm	2.39 mm
FAP	15.32 mm	5.21 mm	10.65 mm	1.96 mm
FDP	7.57 mm	1.84 mm	4.07 mm	1.16 mm
ATH	13.79 mm	5.78 mm	9.91 mm	1.45 mm
ZAS	8.21 mm	2.88 mm	4.99 mm	1.17 mm
ZAT	13.16 mm	7.23 mm	10.32 mm	1.16 mm
TZD	13.10 mm	6.51 mm	9.53 mm	1.38 mm

*Abbreviations*: *max *maximum value, *min *minimum value, *mean *mean value, *sd *standard deviation, *CML *condyle medial-lateral, *CAP *condyle anterior-posterior, *CPH *condylar process height, *ICA *intercondylar angle, *RCR *ramus-condyle ratio, *FML *fossa medial-lateral, *FAP *fossa anterior-posterior, *FDP *fossa´s deepest point, *ATH *articular tubercle height, *ZAS *zygomatic arch root sagittal, *ZAT *zygomatic arch root transverse, *TZD *tubercle-zygomatic arch root sagittal distance

Sex-based comparisons revealed significant differences in six of the twelve TMJ parameters. For the mandibular condyle unit, CML was significantly larger in men than in women (*p* < 0.001). For the glenoid fossa unit, FAP was significantly larger in women (*p* = 0.034). In men, ATH was significantly higher (*p* = 0.003), and ZAS and ZAT were larger (*p* = 0.001 and *p* < 0.001, respectively) (Table 2).

**Table 2:** Sex-related measurement of TMJ parameters

	Male			Female			
Mandibular condyle unit	*n*	mean	sd	*n*	mean	sd	*p*-value
CML	58	21.36 mm	1.81 mm	39	19.55 mm	1.84 mm	*< 0.001*
CAP	58	10.92 mm	1.52 mm	39	10.47 mm	1.01 mm	*ns*
CPH	58	19.27 mm	2.93 mm	39	18.39 mm	2.37 mm	*ns*
ICA	58	135.88 °	11.47 °	39	137.62 °	15.51 °	*ns*
RCR	58	66.81%	3.08%	39	67.64%	3.12%	*ns*
Glenoid fossa unit	*n*	*mean*	*sd*	*n*	*mean*	*sd*	*p-value*
FML	58	19.84 mm	2.72 mm	39	19.60 mm	1.84 mm	*ns*
FAP	58	10.30 mm	2.03 mm	39	11.17 mm	1.76 mm	*< 0.034*
FDP	58	4.03 mm	1.08 mm	39	4.14 mm	1.28 mm	*ns*
ATH	58	10.26 mm	1.36 mm	39	9.39 mm	1.45 mm	*= 0.003*
ZAS	58	4.68 mm	1.02 mm	39	5.45 mm	1.24 mm	*= 0.001*
ZAT	58	10.90 mm	1.01 mm	39	9.44 mm	1.03 mm	*< 0.001*
TZD	58	9.83 mm	1.37 mm	39	9.07 mm	1.27 mm	*= 0.007*

*Abbreviations*: *mean *mean value, *sd *standard deviation, *ns *not significant, *CML *condyle medial-lateral, *CAP *condyle anterior-posterior, *CPH *condylar process height, *ICA *intercondylar angle, *RCR *ramus-condyle ratio, *FML *fossa medial-lateral, *FAP *fossa anterior-posterior, *FDP *fossa´s deepest point, *ATH *articular tubercle height, *ZAS *zygomatic arch root sagittal, *ZAT *zygomatic arch root transverse, *TZD *tubercle-zygomatic arch root sagittal distance

Age-related analysis revealed that middle-aged participants showed a significantly larger CAP than young-aged participants (*p* = 0.044), while ZAS was significantly smaller in the young-aged group than in the middle- and old-aged groups (*p* = 0.022). No significant differences concerning age were found for the other TMJ parameters (Table 3).

**Table 3:** Age-related measurement of TMJ parameters

	Young-aged group (18–38 years)			Middle-aged group (39–59 years)			Old-aged group (60–79 years)		
Mandibular condyle unit	*n*	mean	sd	*n*	mean	sd	*n*	mean	sd
CML	47	20.89 mm	1.81 mm	38	20.65 mm	2.21 mm	12	19.62 mm	2.02 mm
CAP	47	10.42 mm	1.37 mm	38	11.15 mm	1.37 mm	12	10.69 mm	0.87 mm
CPH	47	18.77 mm	2.83 mm	38	19.26 mm	2.79 mm	12	18.39 mm	2.20 mm
ICA	47	136.10 °	12.55 °	38	136.50 °	14.37 °	12	138.76 °	12.65 °
RCR	47	66.65%	3.02%	38	67.32%	3.40%	12	68.53%	2.00%
Glenoid fossa unit	*n*	*mean*	*sd*	*n*	*mean*	*sd*	*n*	*mean*	*sd*
FML	47	19.84 mm	2.42 mm	38	20.03 mm	2.36 mm	12	18.47 mm	2.15 mm
FAP	47	10.80 mm	2.10 mm	38	10.55 mm	1.95 mm	12	10.45 mm	1.53 mm
FDP	47	4.08 mm	1.15 mm	38	4.20 mm	1.25 mm	12	3.65 mm	0.85 mm
ATH	47	9.84 mm	1.61 mm	38	10.05 mm	1.39 mm	12	9.73 mm	0.94 mm
ZAS	47	4.66 mm	1.02 mm	38	5.25 mm	1.18 mm	12	5.46 mm	1.40 mm
ZAT	47	10.48 mm	1.25 mm	38	10.26 mm	1.19 mm	12	9.88 mm	1.35 mm
TZD	47	9.75 mm	1.25 mm	38	9.26 mm	1.50 mm	12	9.55 mm	1.39 mm

*Abbreviations*: *mean *mean value, *sd *standard deviation, *CML *condyle medial-lateral, *CAP *condyle anterior-posterior, *CPH *condylar process height, *ICA *intercondylar angle, *RCR *ramus-condyle ratio, *FML *fossa medial-lateral, *FAP *fossa anterior-posterior, *FDP *fossa´s deepest point, *ATH *articular tubercle height, *ZAS *zygomatic arch root sagittal, *ZAT *zygomatic arch root transverse, *TZD *tubercle-zygomatic arch root sagittal distance

**Fig. 4:**
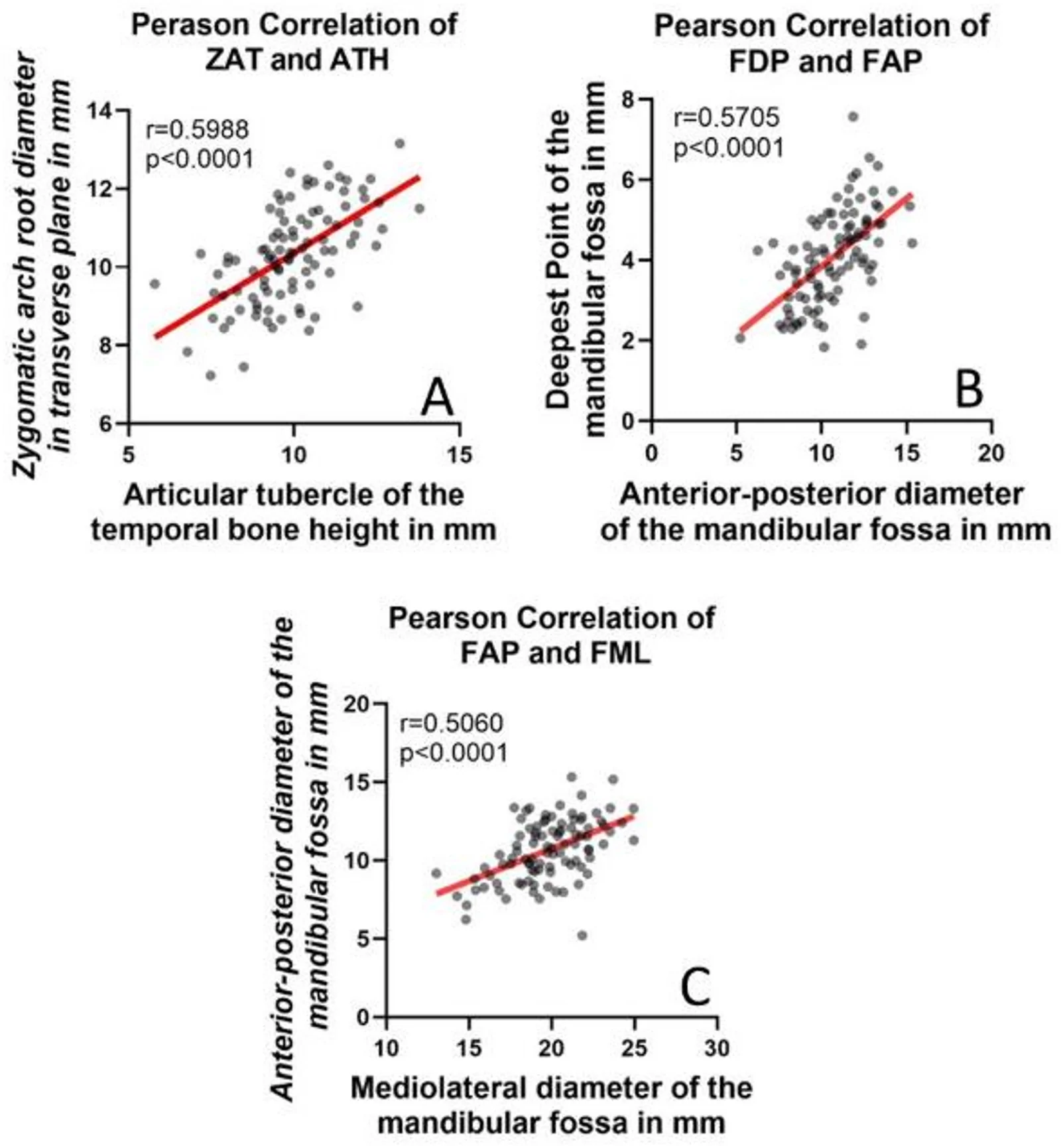
Linear relationship between measured TMJ parameters detected by Pearson correlation. **A** Zygomatic arch root transverse (ZAT) and articular tubercle height (ATH). **B** Fossa´s deepest point (FDP) and fossa anterior-posterior (FAP). **C** Fossa anterior-posterior (FAP) and fossa medial-lateral (FML). Pearson correlation coefficients (*r)* and *p-*values are shown

Correlation analysis revealed the strongest linear relationship between ZAT and ATH (*r* = 0.5899; *p* < 0.0001) (Fig. 4A**)**. However, significant correlations were also found between FML and FDP (*r* = 0.5705; *p* < 0.0001) as well as FML and FAP (*r* = 0.5060; *p* < 0.0001) (Fig. 4B and C**).** No strong correlations were observed for the remaining TMJ parameters (Supplementary file 1).

## Discussion

The novelty of the present study should be interpreted in methodological rather than absolute terms. CT-based morphometric studies of the mandibular condyle and glenoid fossa have previously been published in different populations. However, many of these studies performed measurements directly on radiological datasets or multiplanar reconstructions. In contrast, the present study used CT-derived virtual-digital 3D surface models as the basis for all morphometric measurements. Together with the European cohort and the implant-oriented set of osseous parameters, this represents the specific contribution of the present work. Until now, *Zhang et al.* also applied comparable CT-derived virtual-digital 3D surface model analysis exclusively from an Asian population [16]. Regarding relevant TMJ parameters and subunits, comparison of studies revealed that CAP, ATH, and ZAS showed comparable mean values between the European cohort and the previously reported Asian cohort, although subtle differences were observed (CAP: 10.74 versus 11.23 mm; ATH: 9.91 versus 9.69 mm; ZAS: 4.99 versus 5.73 mm). Notably, mean ATH appeared larger in the European cohort, while mean CAP and ZAS were larger in the Asian cohort [16]. However, differences in craniofacial morphology among populations and inter-ethnic variations have been previously described [17]. The exploratory sex-based comparisons suggested that several TMJ parameters were larger in men than in women, which is consistent with previous observations that craniofacial and mandibular joint dimensions may differ by sex [18]. Interestingly, the present virtual-digital analysis showed that FAP was larger in women. Although this remains to be confirmed in future studies, sex-specific differences in the surrounding soft tissue and muscular anatomy of the TMJ may provide a possible explanation for this finding. In this context, women have been reported to exhibit a higher prevalence of existing TMJ hypermobility, which may potentially lead to remodelling of the glenoid fossa [19]. Furthermore, weakness of the masticatory muscles is more commonly observed in women [20]. Thus, the differences in masticatory muscle strength and individual chewing behaviour may influence joint kinematics over time and may potentially modulate the glenoid fossa´s morphology. Regarding current results of age-related subgroups, most TMJ parameters showed no significant differences. Exploratory differences were observed only for CAP and ZAS, with larger values in the middle-aged subgroup than in the young-aged subgroup. Adaptations due to dental and mandibular loading as well as remodelling processes associated with age-related TMJ disorders may lead to progressive changes of bony TMJ subunits in older individuals in this context [21, 22].

Correlations between ZAT, ATH, FML, and FDP, as well as between FML and FAP, were found, suggesting that some osseous dimensions of the glenoid fossa region may vary in relation to each other. However, the observed correlations were moderate and should not be interpreted as evidence of functional dependence, causal adaptation, or optimized joint biomechanics. Therefore, the correlations reported in the present study should be regarded as descriptive morphometric associations rather than as direct evidence for coordinated functional adaptation. However, from a design perspective, these morphological relationships may nevertheless be relevant as anatomical reference information for future patient-specific TMJ implants, particularly regarding the dimensions of the glenoid fossa subunit [23]. Overall, morphometric reference data may be useful for future implant-related research. The present measurement protocol provides descriptive information on the variability of osseous articulating TMJ subunits in a European cohort and may support future studies that combine anatomy with engineering validation. For example, condylar and glenoid fossa dimensions may inform hypothesis generation for implant sizing concepts, parametric design studies, or comparative analyses against existing stock and patient-specific prosthesis geometries. However, such applications require additional analyses, including clustering or dimensionality reduction, implant simulation, finite element analysis, dynamic mandibular kinematic assessment, and clinical outcome evaluation. Several limitations should be acknowledged. First, the retrospective exploratory design and moderate sample size limit the strength of inference and preclude far-reaching conclusions. In addition, the old-aged subgroup was comparatively small, which may have reduced statistical power for age-related comparisons; age-related findings should therefore be interpreted cautiously. Second, although bilateral averaging was used to avoid treating both TMJs from the same individual as independent observations, this approach may have masked natural side-to-side asymmetry. Such asymmetry may be clinically relevant for patient-specific prosthesis research and should be addressed in future side-specific analyses. Third, all measurements were performed three times by the same observer and mean values were used for analysis. However, repeated measurements and averaging do not establish formal measurement reproducibility. Formal intraobserver reliability statistics, such as intraclass correlation coefficients with 95% confidence intervals, standard error of measurement, or Bland-Altman analysis, were not performed. Therefore, relatively small differences, particularly those with borderline p-values, should be interpreted cautiously and require confirmation in future studies with formal reliability assessment. Fourth, formal testing of normality and homogeneity of variance was not included in the original statistical workflow before applying parametric tests. Therefore, the inferential analyses should be regarded as exploratory rather than confirmatory. Fifth, the analysis was based exclusively on CT-derived osseous morphology. MRI findings and dynamic open- and closed-mouth recordings were not consistently available and could therefore not be included. Accordingly, soft tissue structures, mandibular kinematics, and functional joint behaviour could not be assessed. Sixth, segmentation and surface smoothing may have introduced geometric changes to the reconstructed 3D models, and potential smoothing-related alterations of fine osseous details cannot be fully excluded.

Therefore, the relevance of these morphometric findings for patient-specific TMJ implant research remains exploratory and requires validation in future biomechanical, computational, implant-specific, and clinical studies.

## Conclusion

The present study provides CT-based three-dimensional morphometric reference data on the osseous articulating TMJ subunits in a European cohort. The findings indicate inter-individual variability and exploratory sex- and age-related differences in selected parameters, supporting the concept that mandibular condyle and glenoid fossa morphology vary among individuals. These parameters may be useful as anatomical reference information for future patient-specific TMJ implant research. However, this study was descriptive in nature and did not assess implant fit, mandibular kinematics, prosthesis performance, wear behavior, contact mechanics, or patient outcomes. Therefore, the translational relevance of these morphometric findings requires further validation through biomechanical simulations, dynamic functional analyses, implant-specific design studies, and clinical outcome research.

## Supplementary Information

Supplementary Material 1.

## Data Availability

The data are available from the corresponding author on reasonable request.

## Abbreviations

3D: Three-dimensional
CT: Computed Tomography
TMJ: Temporomandibular joint
DICOM: Digital imaging and communications in medicine
HU: Hounsfield Units
CML: Medio-Lateral diameter of the Mandibular Condyle
CAP: Anterior-Posterior diameter of the Mandibular Condyle
CPH: Condylar Process Height
ICA: Intercondylar Angle
RCR: Ramus-Condyle Ratio
FML: Medio-Lateral diameter of the Glenoid fossa
FAP: Anterior-Posterior diameter of the Glenoid fossa
FDP: Deepest Point of the Glenoid fossa on the FML plane
ATH: Articular tubercle of temporal bone height
ZAS: Diameter of the zygomatic arch root in sagittal plane
ZAT: Diameter of the zygomatic arch root in transverse plane
TZD: Distance between ATH and ZAS
